# Distinct gene mutation profiles among multiple and single primary lung adenocarcinoma

**DOI:** 10.3389/fonc.2022.1014997

**Published:** 2022-12-02

**Authors:** Yadong Wang, Guanghui Wang, Haotian Zheng, Jichang Liu, Guoyuan Ma, Gemu Huang, Qingtao Song, Jiajun Du

**Affiliations:** ^1^ Institute of Oncology, Shandong Provincial Hospital, Shandong University, Jinan, Shandong, China; ^2^ Department of Thoracic Surgery, Shandong Provincial Hospital, Shandong University, Jinan, Shandong, China; ^3^ Research and Development Department, Amoy Diagnostics Co., LTD., Xiamen, Fujian, China

**Keywords:** lung adenocarcinoma, multiple primary lung cancer, multifocal lung cancer, mutation, next generation sequencing

## Abstract

With the development of technologies, multiple primary lung cancer (MPLC) has been detected more frequently. Although large-scale genomics studies have made significant progress, the aberrant gene mutation in MPLC is largely unclear. In this study, 141 and 44 lesions from single and multiple primary lung adenocarcinoma (SP- and MP-LUAD) were analyzed. DNA and RNA were extracted from formalin-fixed, paraffin-embedded tumor tissue and sequenced by using the next-generation sequencing-based YuanSu450TM gene panel. We systematically analyzed the clinical features and gene mutations of these lesions, and found that there were six genes differently mutated in MP-LUAD and SP-LUAD lesions, including RBM10, CDK4, ATRX, NTRK1, PREX2, SS18. Data from the cBioPortal database indicated that mutation of these genes was related to some clinical characteristics, such as TMB, tumor type, et al. Besides, heterogeneity analysis suggested that different lesions could be tracked back to monophyletic relationships. We compared the mutation landscape of MP-LUAD and SP-LUAD and identified six differentially mutated genes (RBM10, CDK4, ATRX, NTRK1, PREX2, SS18), and certain SNV loci in TP53 and EGFR which might play key roles in lineage decomposition in multifocal samples. These findings may provide insight into personalized prognosis prediction and new therapies for MP-LUAD patients.

## Introduction

Lung cancer is still the first cause of oncological death. Lung cancer can be divided into two broad categories according to histology: small-cell lung cancer and non-small-cell lung cancer (NSCLC) which accounts for approximately 85%. subdivided into adenocarcinoma (LUAD; 60%), squamous cell carcinoma (LUSC; 30–35%), large cell carcinoma, and other rare tumors, including adenosquamous carcinoma and in recent years, the ratio of LUAD among NSCLC is rising (1).

With the development of technologies, especially high-resolution computed tomography (HRCT), and the conduction of early lung cancer screening, multiple primary lung cancer (MPLC) has been detected more frequently (2–4). In particularly, adenocarcinoma was shown to be the most common pathological type in MPLC (5).

MPLC is a unique type of lung cancer, defined by presence of at least two independent primary tumors. MPLC was divided into synchronous MPLC (sMPLC) and metachronous MPLC (mMPLC) according to the diagnosis interval (6–8). According to current research, surgical resection was still recommended as the first choice for certain MPLC (9–12). Nowadays, with improved research methods such as machine learning, some studies would combine CT findings and gene sequencing technologies to diagnose and distinguish multiple primary lung cancers from pulmonary metastasis (8, 13–16). Understanding the molecular determinants of MPLC is one of the critical challenges in oncology.

Cancer is a genetic disease. Rapid advancing in next-generation sequencing technology and The Cancer Genome Atlas (TCGA) have profiled and analyzed the molecular aberrations at the DNA, RNA levels (17, 18). Genome instability and mutation is one of the hallmarks of cancer (19, 20). The accumulation of somatic mutations in the DNA affected neoplastic transformation, including driver mutations, mutations that directly affect tumor growth, such as TP53, epidermal growth factor receptor (EGFR) or RAS, and passenger mutations, which do not directly impact the growth of the cancer cell (21, 22).

In lung adenocarcinoma, several large-scale genomics studies have analyzed the genomic mutational landscape and some important targets, including EGFR, ALK, RAS, TP53 were identified to be associated with overall survival or treatments (17, 23–26). Besides, the most common therapeutic targets are EGFR and BRAF mutations and ALK and ROS1 rearrangements (17). Despite this progression, the aberrant gene mutation in MPLC compared with single primary lung cancer (SPLC) is largely unclear.

In this study, we included 141 single primary lung adenocarcinoma (SP-LUAD) patients and 44 multiple primary lung adenocarcinoma (MP-LUAD) patients to analysis the mutational landscape and there was an apparently difference between these two groups. RBM10, CDK4, ATRK, NTRK1, PREX2 and SS18 were identified as the significantly differential gene. The relationship between these genes and clinical characters was conducted using cBioPortal database. The study of associated gene mutations in MPLC will provide new insight into the mechanism, potential therapeutic targets, promote the prognosis and survival in clinic.

## Material and methods

### Patient cohort description

Primary lung cancers were collected between January 2018 and December 2020 in our institution. After sample collection, surgical specimens and biopsy tissues were snap-frozen in liquid nitrogen within 30 minutes of resection. Genomic DNA was extracted from all included samples. 141 SP-LUAD and 44 MP-LUAD patients were included in this study. Among these MP-LUAD specimens, 73 lesions were analyzed, including 9 lesions from 3 three-primary cases, 46 lesions from 23 dual-primary cases and 18 lesions from the other 18 MP-LUAD cases.

MP-LUAD were defined according to Warren and Gate’s criteria: 1) each tumor had to show definite features of malignancy; 2) each cancer had to be anatomically separate and distinct; 3) the possibility that one cancer was a recurrence or metastatic lesion of the first cancer had to be ruled out; and 4) the subsequent primary malignancies had to be present in either the same or different organs (27).

### Tumor processing and DNA extraction

Before DNA extraction, 4 μm formalin-fixed paraffin-embedded (FFPE) specimens were taken. Histopathological examination confirmed that the area of each specimen was greater than 1 cm2 and the tumor cell density was greater than 20%.

According to the manufacturer’s instructions, 0.5-2 μg of cancer tissue DNA was extracted from 4 μm FFPE tumor samples using a DNA extraction kit (QIAamp DNA FFPE tissue kit).

### Library construction

Libraries were constructed using Roche’s KAPA Hyper Prep Kit according to the manufacturer’s instructions. This custom hybrid capture panel includes more than 23,660 individually synthesized 5’-DNA biotin-labeled 120 bp oligonucleotides to target the approximately 2.6 Mb human genome, which contains 7029 extra coding nucleotides of 468 cancer-related genes exons and selected introns of 39 genes that are frequently rearranged in solid tumors.

Hybridization capture was performed according to the protocol of “IDT Company xGen LOCKDOWN probe and reagent hybridization capture DNA library”, and sequenced on Illumina Nextseq500 with an average coverage of 1000 times. According to this method, paired paired-end sequencing (2 × 150 bp) was obtained by OrigiMed (OrigiMed, College of American Pathologists (CAP) and Clinical Laboratory Improvement Amendments (CLIA) accredited laboratory, Shanghai, China) Comprehensive genomic profiling, including single nucleotide variation (SNV), short and long insertion/deletion (INDELS), copy number variation (CNV), gene rearrangements and gene fusions.

### Next-generation sequencing

DNAs of both FFPE tumor tissues and matched blood were obtained by using QIAamp DNA FFPE Tissue Kit and QIAamp DNA Blood Midi Kit (Qiagen, Hilden, Germany), respectively, and sequenced by using the next-generation sequencing-based YuanSu450TM gene panel of OrigiMed (Shanghai, China), from where the laboratory was certified by CAP and CLIA. The genes were captured and sequenced with a mean depth of 800× by using Illumina Nova (Illumina, Inc., CA).

### Mutation analysis

Genomic alterations were identified as following: SNVs were identified by MuTect (v1.7). Insertion-deletions (Indels) were identified by using PINDEL (V0.2.5). The functional impact of genomic alterations was annotated by SnpEff3.0. CNV regions were identified by Control FREEC (v9.7) with the following parameters: window = 50 000 and step = 10 000. Gene fusions were detected through an in house developed pipeline. Gene rearrangements were assessed by Integrative Genomics Viewer (IGV). Known somatic mutations in the Catalog of Somatic Mutations in Cancer (COSMIC) and known germline polymorphisms in the U.S. National Center for Biotechnology Information’s Single Nucleotide Polymorphism Database (dbSNP) were not counted. Tumor mutation burden (TMB) was calculated by counting the coding somatic mutations, including SNVs and Indels, per megabase of the sequence examined in each patient. The waterfall chart was drawn using the R report “ComplexHeatmap (version=2.2.0)”, and the difference in gene mutation frequency between single foci and multiple foci was compared using the fisher test.

### Phylogenetic analysis

The phylogenetic analysis was conducted using LICHeE as reported before (28). Briefly, the branch evolution of the tumor within each patient was inferred by comparing the list of mutations in each tumor region. Regions containing all mutations observed in another region are indicated as their ancestors. If no such region exists, putative precursors are inferred from a set of changes common to multiple regions. Regions that did not change were considered to be parallel branches, although alternative dendrograms could be formed by assuming that these regions are ancestors of regions with mutations.

### Data source

The mutated gene data was recruited from cBioPortal database (http://www.cbioportal.org), originally developed at Memorial Sloan Kettering Cancer Center, an open-access resource for the interactive exploration of multidimensional cancer genomics data sets (29). The database can provide visualization (the associations between genes and clinical characteristics), analysis and downloads of large-scale cancer genomics data sets (the different expressed genes data). The somatic mutation data was acquired from the Cancer Genome Atlas (TCGA) database (https://cancergenome.nih.gov/).

### Gene set enrichment analysis

Gene Set Enrichment Analysis (GSEA) was used to analyze the correlation between gene differential expression with cellular pathway (KEGG pathways) and cellular functions (GO-molecular function). “clusterProfiler”, “org.Hs.eg.db”, “enrichplot”, “ggplot2” R packages were applied to perform GSEA and “maftools” for co-occurrence of gene mutations. Adjusted P value < 0.05 was considered as statistically significant.

### Statistical analysis

All tests were performed with the R environment version 4.0.2 (Vienna, Austria), SPSS 25.0 (Chicago, US) or GraphPad Prism 7.0 (San Diego, CA). Comparisons of clinical characteristics between paired primary tumors and metastases were based on Student’ s t test, Chi-square test and Fisher’s exact test. The nonparametric Wilcoxon rank-sum test was applied for comparison of mutation counts and branch lengths. If not noted otherwise, the tests applied were two-sided. As per the convention, p<0.05 was considered statistically significant.

## Results

### Patient characteristics

There were 141 SP-LUAD patients and 44 MP-LUAD patients in the study. **Table 1** listed all of the patients’ information. There were 86 males and 99 females in this study, with an average age of 57.7 and 59.3 years for the SP- and MP-LUAD cohorts, respectively. Weight Loss was a statistically significant factor across all these factors, indicating that weight loss may be more common in MP-LUAD. Furthermore, each lesion in these cohorts had a different histologic class. In the MP-LUAD cohort, the ratios of lepidic, adenocarcinoma *in situ* (AIS), and microinvasive adenocarcinoma (MIA) were higher, while acinar, solid, papillary, and micropapillary were lower.

**Table 1:** Clinical characteristics of all patients.

Characteristics	SP-LUAD		MP-LUAD		p
	n.	Ratio	n.	Ratio	
**Gender**					
Male	68	0.482	18	0.409	0.396
Female	73	0.518	26	0.591
**Age**	57.7 ± 10.336		59.32 ± 6.653		0.3308
**TFD**					
<6	104	0.738	30	0.682	0.470
≥6	37	0.262	14	0.318
**Symptom**					
Pos.	50	0.355	13	0.295	0.470
Neg.	91	0.645	31	0.705
**Chest Discomfort**					
Pos.	24	0.170	11	0.250	0.238
Neg.	117	0.830	33	0.750
**Weight Loss**					
Pos.	6	0.043	6	0.136	0.027
Neg.	135	0.957	38	0.864
**History of Other Tumor**					
Pos.	9	0.064	2	0.045	1
Neg.	132	0.936	42	0.955
**Chronic Disease**					
Pos.	68	0.482	24	0.545	0.464
Neg.	73	0.518	20	0.455
**History of Surgery**					
Pos.	52	0.369	13	0.295	0.374
Neg.	89	0.631	31	0.705
**History of Trauma**					
Pos.	7	0.050	3	0.068	0.704
Neg.	134	0.950	41	0.932
**Allergic History**					
Pos.	11	0.078	6	0.136	0.242
Neg.	130	0.922	38	0.864
**Marriage**					
Married	139	0.986	41	0.932	0.088
Widowed	2	0.014	3	0.068
**Family History of Cancer**					
Pos.	17	0.121	8	0.182	0.300
Neg.	124	0.879	36	0.818
**Family History of Lung Cancer**					
Pos.	11	0.078	5	0.114	0.463
Neg.	130	0.922	39	0.886

Pos., Positive; Neg., Negative; TFD, Time to the First Discovery.

**Figure 1:**
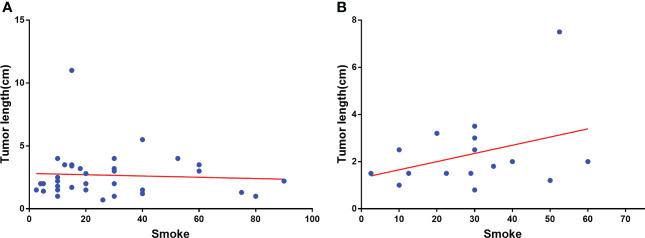
Correlation analysis of smoking amount with the largest lesion diameter of SP-**(A)** and MP-LUAD **(B)**.

In **Table 2**, the right upper lobe lesion accounted for the majority of the cases. There appeared to be no link between smoking or drinking habits and these two types of patients (**Table 3**), and the amount of smoking packs (daily amount of cigarettes*years of smoking/20) was barely correlated with the largest lesion diameter of MP-LUAD (R square: 0.125) (**Figure 1**).

**Table 2:** Pathological correlation analysis.

Characteristics	SP-LUAD		MP-LUAD		p
	n.	Ratio	n.	Ratio	
**Length**	2.45035 ± 1.39988		2.17954 ± 1.2159		0.252
**Subtype**					
Acinar	80	0.567	33	0.452	<0.0001
Solid	14	0.099	1	0.014
Lepidic	12	0.085	13	0.178
Papillary	8	0.057	3	0.041
Micropapillary	6	0.043	2	0.027
Invasive Mucinous	4	0.028	0	0.000
Poorly Differentiated	1	0.007	2	0.027
AIS	1	0.007	4	0.055
IA	2	0.014	9	0.123
MIA	5	0.035	6	0.082
LUAD	8	0.057	0	0.000
**Site**					
Right Upper Lobe	51	0.362	31	0.425	0.085
Right Middle Lobe	8	0.057	10	0.137
Right Lower Lobe	22	0.156	10	0.137
Left Upper Lobe	27	0.191	14	0.192
Left Lower Lobe	33	0.234	8	0.110

**Table 3:** Clinical correlation analysis of smoking and drinking.

Characteristics	SP-LUAD		MP-LUAD		p
	n.	Ratio	n.	Ratio	
**Smoke**					
Ever	13	0.092	3	0.068	0.668
Smoker	29	0.206	12	0.273
Never	99	0.702	29	0.659
**Drink**					
Ever	2	0.014	1	0.023	0.816
Regular	23	0.163	5	0.114
Sometimes	26	0.184	8	0.182
Never	90	0.638	30	0.682

### Mutation profile of the SP-LUAD and MP-LUAD cohort

**Figure 2:**
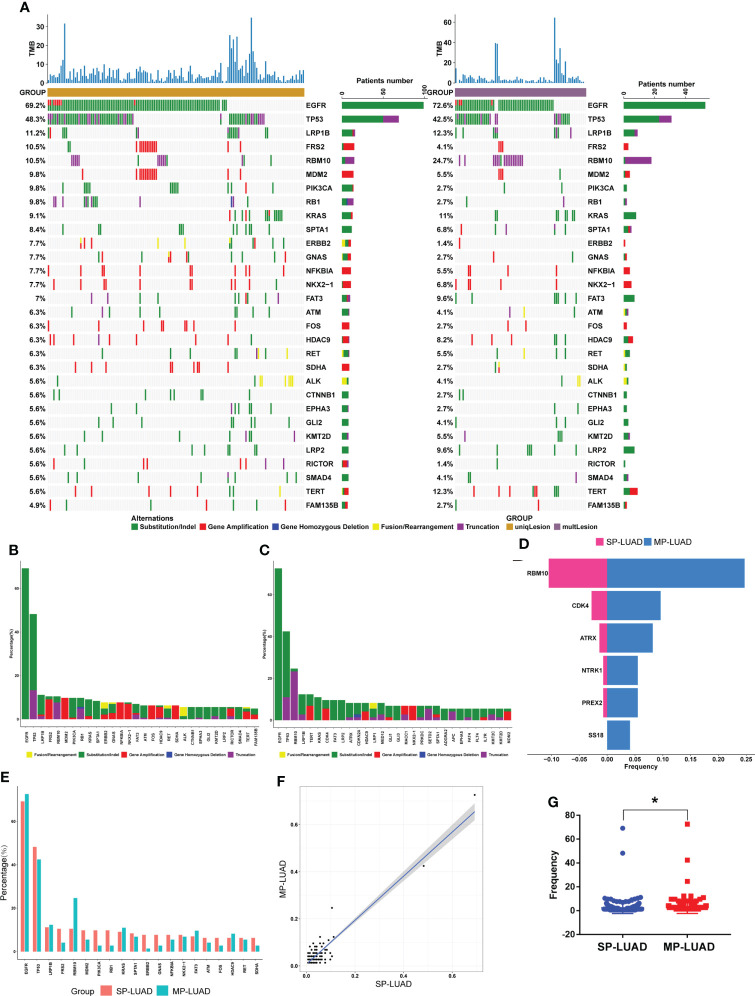
Comparation of SP- and MP-LUAD lesions. **(A)**The waterfall plot of tumor somatic mutation established based on the SP-LUAD (left) and MP-LUAD (right) cohort. **(B, C, E)** The difference of mutation frequency of common mutated genes between SP- and MP-LUAD lesions. **(D)** Six genes statistically different mutated among these two cohorts, including RBM10, CDK4, ATRX, NTRK1, PREX2, SS18. **(F, G)** Mutation Frequency of SP- and MP-LUAD lesions. * p<0.05.

We aimed to analyzed the mutational landscape in the SP-LUAD and MP-LUAD cohort and identified the top 30 most frequently mutant genes in SP-LUAD cohort, including EGFR, TP53, LRP1B, FRS2, RBM10, MDM2, PIK3CA, RB1, KRAS, SPTA1, ERBB2, GNAS, NFKBIA, NKX2-1, FAT3, ATM, FOS, HDAC9, RET, SDHA, ALK, CTNNB1, EPHA3, GLI2, KMT2D, LRP2, RICTOR, SMAD4, TERT, FAM135B (**Figure 2**).

The detail of each altered gene was shown in **Figure 2A**. Apparently, the ratio of each mutated gene in MP-LUAD cohort was not consistent with those in SP-LUAD cohort (**Figures 2B, C**). Then we compared the frequency between these two cohorts, and six genes (RBM10, CDK4, ATRX, NTRK1, PREX2, SS18) were shown to have significantly different mutation frequencies (p=0.0089, 0.0469, 0.0191, 0.0456, 0.0456, 0.0375, respectively) (**Figures 2D, E**). The frequency of two cohorts was shown in **Figures 2F, G**. The MP-LUAD cohort had a higher mutation frequency.

**Figure 3:**
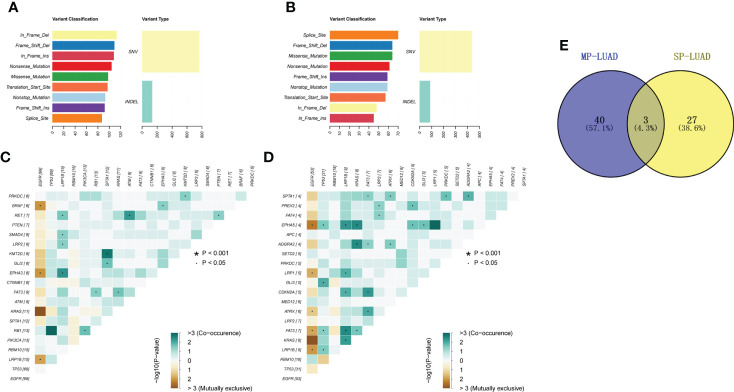
**(A, B)** Mutation types of SP-**(A)** and MP-LUAD **(B)** lesions. **(C, D)** Co-mutation analysis of SP-**(C)** and MP-LUAD **(D)** lesions. **(E)** The common co-mutated genes among SP- and MP-LUAD lesions. * p<0.05.

The gene variant type was shown in **Figure 3A, B**. In the MP-LUAD cohort, the Splicing Site variant was the most common while in frame deletion in the SP- LUAD cohort. **Figures 3C, D** shows the co-occurrence of mutant genes. The co-occurrence signature in MP-LUAD differed from that in SP-LUAD. Only three genetic changes were found to be prevalent (**Figure 3E**). The co-mutated genes of the SP- and MP-LUAD cohorts in EGFR-, KRAS-, and TP53-mutant patients were almost completely different, as shown in **Supplementary Table 1**.

### The associations between the six different mutated genes and patients’ clinical characteristics

**Figure 4:**
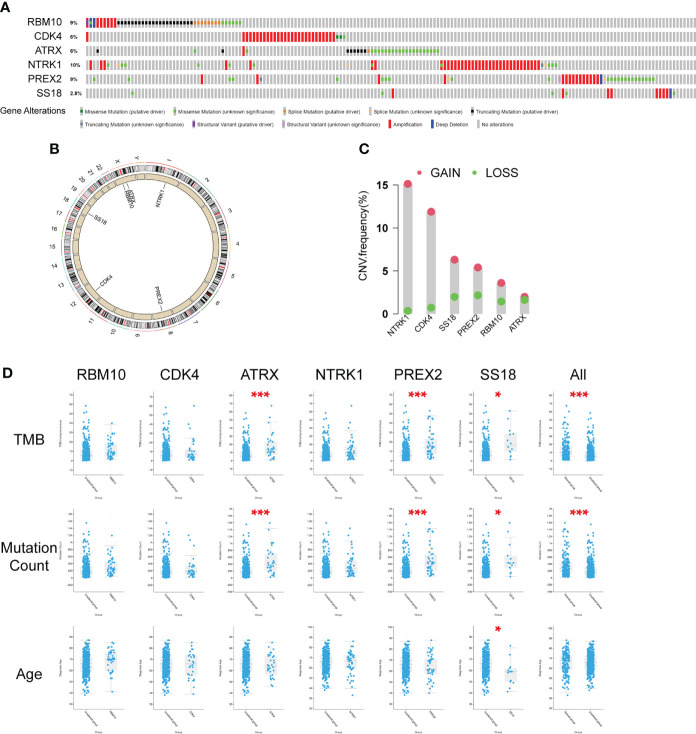
Characteristics of the six differently mutated genes. **(A)** Gene alterations obtained from cBioPortal database. **(B)** Gene location on chromosomes. **(C)** Gene CNV analysis using TCGA data. **(D)** Clinical correlation analysis of six genes using cBioPortal database. * p<0.05, *** p<0.001.

To further investigate the characteristics of these six genes in the public database, we analyzed the data from the cBioPortal database and TCGA database, including the genetic alteration ratio(**Figure 4A**), gene location on chromosomes(**Figure 4B**), the CNV alteration frequency(**Figure 4C**) and the clinical correlations (**Figure 4D**).The investigation of CNV alteration frequency showed most were focused on the amplification in copy number, among which the NTRK1 and CDK4 had a higher frequency(**Figure 4C**). The parameters (Histology, Sex, Tumor type, TMB, Mutation Count, Age) were included (**Figure 4D** and **Supplementary Figure 1**) and the results suggested that TMB (p<0.001) and mutation count (p<0.001) in six-gene altered group was much higher. The single gene analysis of ATRX (p<0.001, p<0.001), PREX2(p<0.001, p<0.001) and SS18(p<0.001, p<0.001) got the similar results. SS18 was unique among the genes studied since it was the only one in which the mutation status was linked to Tumor Type (p=0.0137). There were differences in sex distribution between the mutated and non-mutated groups, but the differences between the two groups were not statistically significant (p=0.397, 0.628, 0.0866, 0.945, 0.602, 0.865). Apart from that, the mutations of PREX2 (p=0.0156) and SS18 (p=0.0199) were linked to Fraction Genome Alteration and Ragnum Hypoxia Score, respectively (**Supplementary Figures 1C, D**). However, we found that these genes were unrelated to the patients’ overall survival (**Supplementary Figure 2**).

### Gene expression differences associated with six gene mutation.

**Figure 5:**
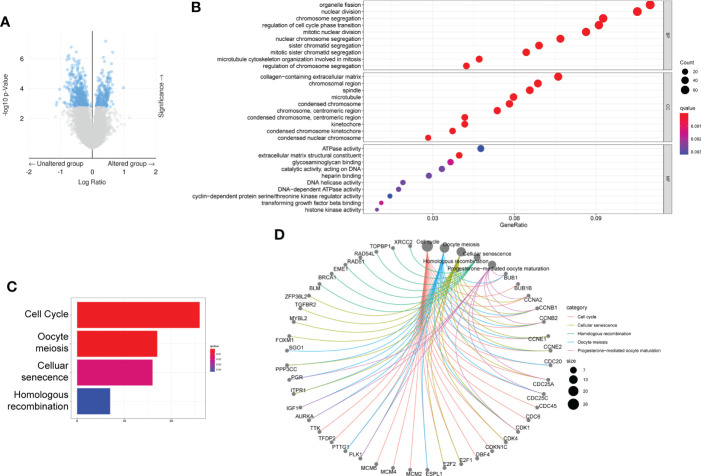
Differential expressed gene based on the mutation status of the six genes. **(A)** Volcano figure of differently expressed genes from cBioPortal database. **(B–D)** GO **(B)** and KEGG **(C, D)** analysis based on the differently expressed genes.

To investigate the Gene expression differences associated with six gene mutation, data from cBioPortal database was recruited, and the volcano plot of differentially expressed genes was shown in **Figure 5A**. Besides, based on these differentially expressed genes, GSEA was performed using R tool. The most enriched KEGG pathways and GO-molecular function terms were summarized in **Figures 5B–D** and the analysis of single gene was shown in **Supplementary Figures 3, 4**.

### Heterogeneity of MP-LUAD and phylogenetic reconstruction of MP-LUAD

**Figure 6:**
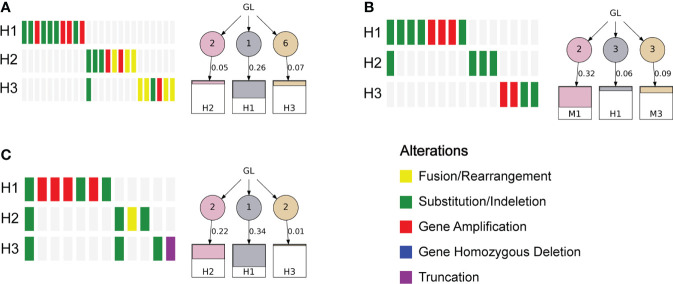
Gene alterations and polygenetic tree of the triple-primary LUAD lesions.

**Figure 7:**
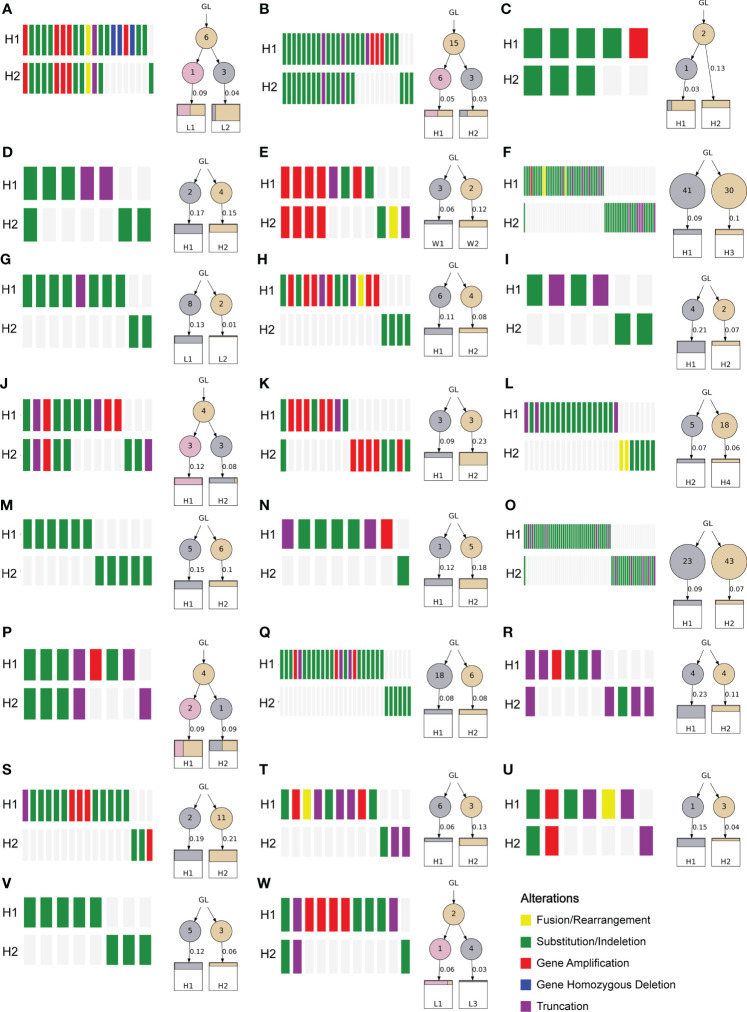
Gene alterations and polygenetic tree of the dual-primary LUAD lesions.

To investigate the heterogeneity of different lesions of MP-LUAD, we performed heterogeneity analysis. The percentage of mutated genes in each lesion was summarized in **Figures 6**, **7**.

As we could see that distinct lesions from one patient were virtually entirely different. Only one case, who carried three lesions, shared one SNV, EGFR L858R, as shown in **Figure 6C**, while the others were completely distinct (**Figures 6A, B**). The phylogenetic trees of all two-lesion-cases listed in **Figure 7** indicated that there were still some relationships among two lesions in most cases. The number in the circle denoted the number of SNVs that had been adjusted, while the number near the arrow signified the SNVs’ contributions. Monophyletic connections might be traced back to several lesions. We evaluated the contribution value and amino acid changes associated with these genes and discovered that five TP53 and EGFR mutations were the most significant (**Supplementary Table 2**).

## Discussion

MPLC has become increasingly common as medical technology has advanced, but despite recent advances in large-scale genomics investigations, little is known about its gene signature, which remains a major problem of therapy failure and poor long-term survival.

In the present study, we conducted a mutation analysis and compared the difference between MP-LUAD and SP-LUAD. Obviously, the frequency of each mutated gene in MPL-LUAD cohort was not consistent with these in SP-LUAD cohort The related clinical characteristics was shown in **Table 1**. It seemed that while there were no significantly difference of age, chest discomfort and family history between these two groups, MP-LUAD patients were related to a higher ratio of weight loss (**Table 1**). One of the clinical characteristics called Time to the First Discovery (TFD) attracted us, which reflected the length of medical history. Although there was no statistical difference of TFD, probably limited by sample size, MP-LUAD patients showed little symptoms (cough, expectoration, and hemoptysis) which might reduce their willingness to further therapy and showed a longer TFD (**Table 1**), and this result might suggest that we had to pay more attention to TFD.

The amount of smoking was barely correlated with the diameter in MP-LUAD patients (**Figure 1B**). The TMB in the MP-LUAD cohort (3.381 ± 0.3586) was greater than the SP-LUAD cohort (2.434 ± 0.2615), according to our findings (**Figure 2G**).

Importantly, we found there were 6 genes with significantly different mutation frequency among these patients, including RBM10, CDK4, ATRX, NTRK1, PREX2, SS18. It was obviously that the mutation frequency of RBM10 in MP-LUAD cases was the highest and followed by CDK4, ATRX, NTRK1, PREX2, SS18.

RBM10 (RNA-binding motif protein 10), a member of the RNA-binding protein (RBP) family, is located at p11.3 on the X chromosome and also an alternative RNA splicing factor that participates in the regulation of gene expression (30). Recently, RBM10 was reported to function as an oncogene in LUAD by activating EGFR, mitogen-activated protein kinase (MAPK) and phosphoinositide 3-kinase (PI3K)-AKT pathways and inhibition of apoptotic pathways (31), consist with the result in this study shown in **Figure 5C** and **Supplementary Figure 3**. Apart from that, Zhao, Jiawei et al. found that RBM10 mutations contributed to lung adenocarcinoma pathogenesis by deregulating splicing (32). Vinayanuwattikun, Chanida et al. revealed that the number of RBM10 mutations was higher in invasive lung adenocarcinoma (33), which suggested that RBM10 contributed to the LUAD progression and might also explain the higher mutation frequency of RBM10 in MP-LUAD.

CDK4 (Cyclin-dependent kinase 4) is a well-recognized cyclin-dependent kinase that specifically regulate cellular transition from the G1 phase to S phase of cell cycle together with CDK6 (34–36). As **Figure 4A** shows, the most frequent alteration of gene CDK4 is Amplification. CDK4 Amplification was seen in several tumors, such as head and neck mucosal melanoma (37), urinary bladder cancer (38), liposarcomas (39), melanoma (40) and lung cancer (41–43). Dysregulation of the cyclin D–CDK4/6–INK4–Rb pathway results in increased proliferation and due to the importance of CDK4/6 activity in cancer cells, CDK4/6 inhibitors seem as promising treatment (44). However, CDK4 amplification may reduce sensitivity to CDK4/6 inhibition in some cases (45). As **Supplementary Figure 3** showed, mutation of gene CDK4 was related to “regulation of response to drug”, and it was reported that in lung cancer, amplification of CDK4 was significant in *de novo* EGFR TKI resistance (43).

Gene ATRX (Alpha thalassemia/mental retardation syndrome X-linked chromatin remodeler) was first discovered in the X-linked mental retardation syndrome (ATRX syndrome) patients (46). And nowadays, its role in cancer is emerging. Araujo-Castro Marta et al. reported that ATRX mutation was linked to a shorter disease-specific survival (47). ATRX protein is one of the SWI/SNF2 (SWItch/Sucrose Non-Fermentable) family of chromatin remodeling proteins, and maintains genomic stability through its deposition of the replication-independent histone variant H3.3 at telomeres and pericentromeric heterochromatin (48). As shown in **Figure 4A**, Nonsense Mutation of ATRX was the most frequent, and ATRX loss has been shown to promote ALT, DNA damage and replicative stress (49–51). The GSEA analysis indicated that mutation of gene ATRX was related to “humoral immune response” (**Supplementary Figure 3**), and now it has been reported in many tumors including pleomorphic Sarcomas (52), glioma (53, 54), gastric cancer patients (55), while the relationship between mutation of ATRX with cytochrome P450 was not reported.

NTRK1 (Neurotrophic receptor tyrosine kinase 1) was originally identified as a fusion oncogene, trkA (tropomyosin receptor kinase) (56). The NTRK1 gene belongs to nerve growth factor receptor genes family, which mainly expressed in neuronal system (57). Several fusion partner genes of NTRK1 were reported in the past few years in thyroid cancer, glioblastoma and lung cancer (58). Many drugs have been developed for the treatment of NTRK1-rearanged cancers. However, it is worth noting that fusions of the NTRK1 genes with CD74 and MPRIP genes were identified in only 3% of some American patients and none of others (59). In this study, we identified two NTRK1 gene alterations including Gene Amplification (1.37% in MP-LUAD) and Substitution (0.7% and 4.11% in SP- and MP-LUAD, respectively) (**Figures 2B, C**) and it showed a highest gene alteration ratio and CNV frequency in the public database (**Figures 4A, C**). However, in the clinical correlation analysis, NTRK1 alteration was shown to be statistically unrelated to any clinical factor (**Figure 4D**). Mutations of gene NTRK1 were also reported in lung cancer (60, 61), while the probable mechanism how these mutations excluding Rearrangement mutation contributed to lung cancer progression was still unknown.

PREX2 (Phosphatidylinositol 3,4,5-trisphosphate-dependent Rac exchanger 2) is considered to be an oncogene for the PREX2 protein’ inhibition of phosphatase and tensin homolog (PTEN) and thus activation of PI3K signaling pathway (62–64). The somatic mutation of PREX2 has been reported in several cancers including hepatocellular cancer (65), breast cancer (66), Melanoma (67, 68) and lung cancer (69). In the study of hepatocellular cancer, most mutant forms of PREX2, had an extended half-life compared with wild-type PREX2, and mutated PREX2 also promoted migration and activated the AKT pathway (65). In lung cancer, PREX2 played an important role in mediating the activation of PI3K/Akt signaling pathway (64), which might provide the evidence for the higher mutation frequency in MP-LUAD cases.

SS18 is the only gene that alternated in MP-LUAD (2 and 1 cases for Rearrangement and Amplification, respectively) rather than SP-LUAD. Fusion of SS18 was frequently detected in synovial sarcoma (70–73). In the GSEA analysis, SS18 was shown to be associated with nuclear division and cell adhesion molecules (**Supplementary Figure 3**) indicating that SS18 might promote proliferation and metastasis of cancer cells. Knowledge about the actual oncogenic signals effected by SS18-fusion protein in lung cancer is still limited. In the study of synovial sarcoma, the SS18-SSX fusion protein would induce aberrant YAP/TAZ signals (71) and associated with SWI/SNF and Polycomb chromatin complexes to dysregulate gene expression (74–76), which might provide ideas for the aberrant alternation of SS18 in MP-LUAD.

Co-occurrence of genetic abnormalities was found to impact the response of lung cancer to several anticancer therapy (77). There were 40 co-occurring genomic changes in MP-LUAD patients and only three in common, as shown in **Figure 3E** and **Supplementary Table 1**. In the MP-LUAD cohort, the Splicing Site variation was the most common. The co-mutated genes in EGFR-, KRAS-, and TP53-mutant LUAD were virtually entirely different, as indicated in **Supplementary Table 1**. KRAS and LRP1B were all mutational exclusivity in EGFR-mutated LUAD patients, whereas genes like ATRX, EPHA5, and LRP1 were enriched in MP-LUAD patients. LRP1B was shown to be co-mutated in TP53- and KRAS-mutated MP-LUAD patients, which is comparable to a previous finding (78). Besides, LRP1B was reported to be associated with outcomes to immune checkpoint inhibitors, especially co-mutation of FAT3 and LRP1B in LUAD (79, 80).

Finally, we performed an analysis of the multiple lesions from MP-LUAD patients. According the results in **Figures 6**, **7**, through comparing the contribution value and amino acid variations, we discovered that TP53 and EGFR were the most significantly different mutated genes that might contribute to the oncogenesis of MP-LUAD, and that certain SNV loci in TP53 (F143S, H193R) and EGFR (L747 P753 delinsS, S768 D770dup, T790M) might play key roles in lineage decomposition in multifocal samples (**Supplementary Table 2**).

The present study has several limitations. First, the sample size is too small, which may lead us to neglect some factors; Second, not all the samples were fresh-frozen and we could not guarantee the purity of these samples even though with a strict pathologic evaluation; Third, some MP-LUAD patients who did not undergo surgical treatment were not included, which might lead to selection and information biases.

## Conclusion

In conclusion, we conducted a mutation analysis of SP- and MP-LUAD patients and identified genomic alterations and evolutionary trajectories underlying MP-LUAD. These findings will provide new insights into the oncogenesis of MP-LUAD and useful information for development novel approach to target MP-LUAD. Nevertheless, further research is required to elucidate the functions of these genes and their pathways.

## Data availability statement

The original contributions presented in the study are included in the article/**Supplementary Material**. Further inquiries can be directed to the corresponding author.

## Ethics statement

The studies involving human participants were reviewed and approved by The ethics committees of Shandong Provincial Hospital(No. 2022-159). The patients/participants provided their written informed consent to participate in this study.

## Author contributions

JD contributed to the designing and supervising the study, and correspondence. Co-authors YW and GW analyzed the data and completed the manuscript. HZ, JL, GM, GH, QS helped to collect specimens, search for references and offered guidelines of statistical methods. All authors contributed to the article and approved the submitted version.

## Funding

This work is supported by Shandong Provincial Natural Science Foundation (Grant No. ZR2021MH192 and ZR2020QH215).

## Acknowledgments

Thanks for everyone who helped to complete this study.

## Conflict of interest

Authors GH and QS are employed by Amoy Diagnostics Co., LTD.

The remaining authors declare that the research was conducted in the absence of any commercial or financial relationships that could be construed as a potential conflict of interest.

## Publisher’s note

All claims expressed in this article are solely those of the authors and do not necessarily represent those of their affiliated organizations, or those of the publisher, the editors and the reviewers. Any product that may be evaluated in this article, or claim that may be made by its manufacturer, is not guaranteed or endorsed by the publisher.

## Abbreviations

NSCLC, non-small-cell lung cancer; LUAD, adenocarcinoma; LUSC, squamous cell carcinoma; HRCT, high-resolution computed tomography; MPLC, multiple primary lung cancer; SPLC, single primary lung cancer; TCGA, The Cancer Genome Atlas; EGFR, epidermal growth factor receptor; MP-LUAD, multiple primary lung adenocarcinoma; SP-LUAD, single primary lung adenocarcinoma; FFPE, formalin-fixed paraffin-embedded; CAP, College of American Pathologists; CLIA, Clinical Laboratory Improvement Amendments; SNV, single nucleotide variation; INDELS, short and long insertion/deletion; CNV, copy number variation; IGV, Integrative Genomics Viewer; TMB, Tumor mutation burden; GSEA, Gene Set Enrichment Analysis; TFD, time to the first discovery; AIS, adenocarcinoma in situ; MIA, microinvasive adenocarcinoma; RBP, RNA-binding protein; MAPK, mitogen-activated protein kinase; PI3K, phosphoinositide 3-kinase; RBM10, RNA-binding motif protein 10; CDK4, Cyclin-dependent kinase 4; ATRX, Alpha thalassemia/mental retardation syndrome X-linked chromatin remodeler; NTRK1, Neurotrophic receptor tyrosine kinase 1; PREX2, Phosphatidylinositol 3,4,5-trisphosphate-dependent Rac exchanger 2; PTEN, phosphatase and tensin homolog.
