# Supplementary material for: Distinct gene mutation profiles among multiple and single primary lung adenocarcinoma
Source: Front Oncol. 2022 Dec 2;12:1014997. doi: 10.3389/fonc.2022.1014997 (PMC9755731; doi:10.3389/fonc.2022.1014997)
Supplement: Supplementary file 2 [file DataSheet_2.pdf]

Supplementary Figure

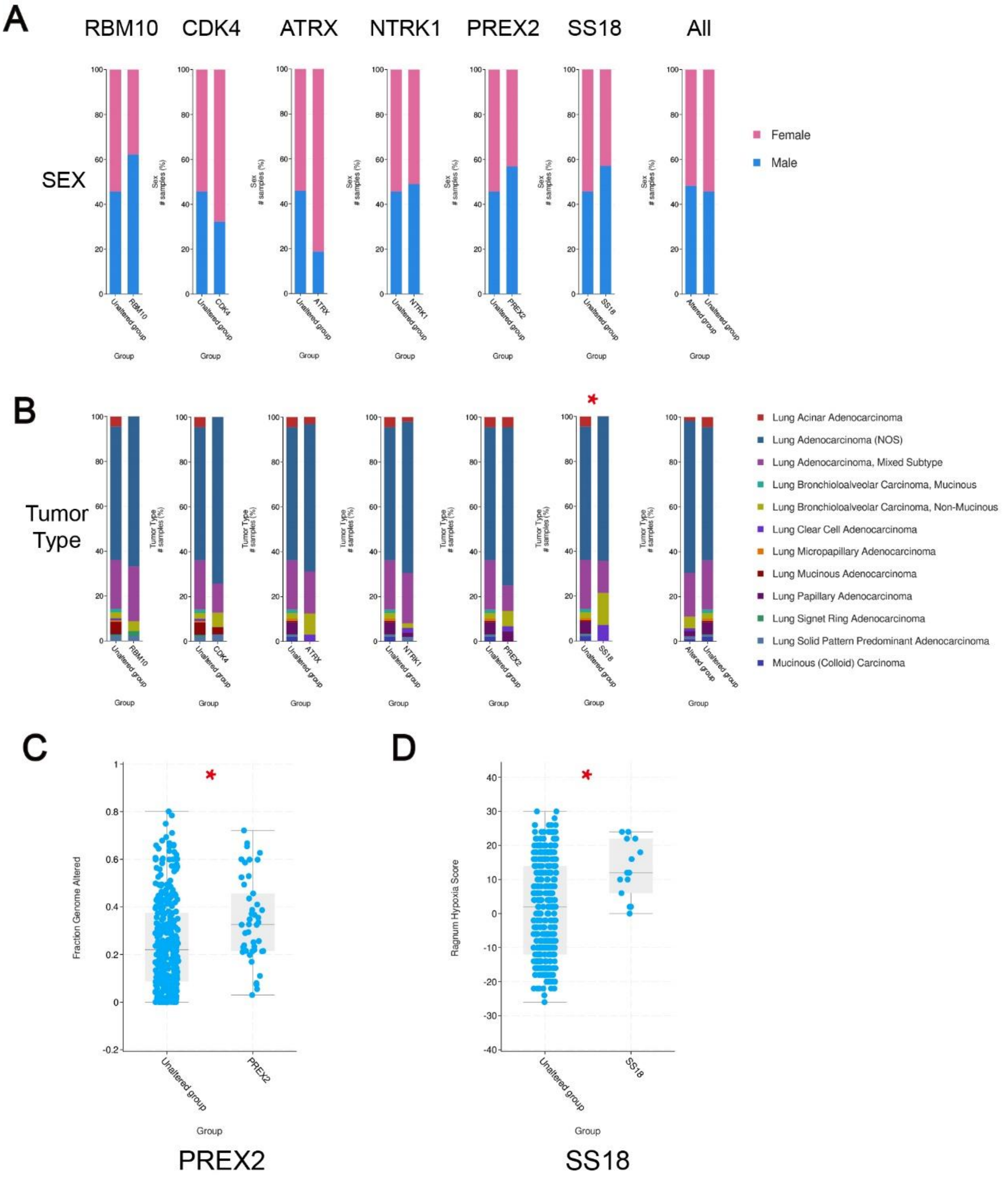

Fig.1 Clinical correlation analysis of six genes using cBioPortal database.

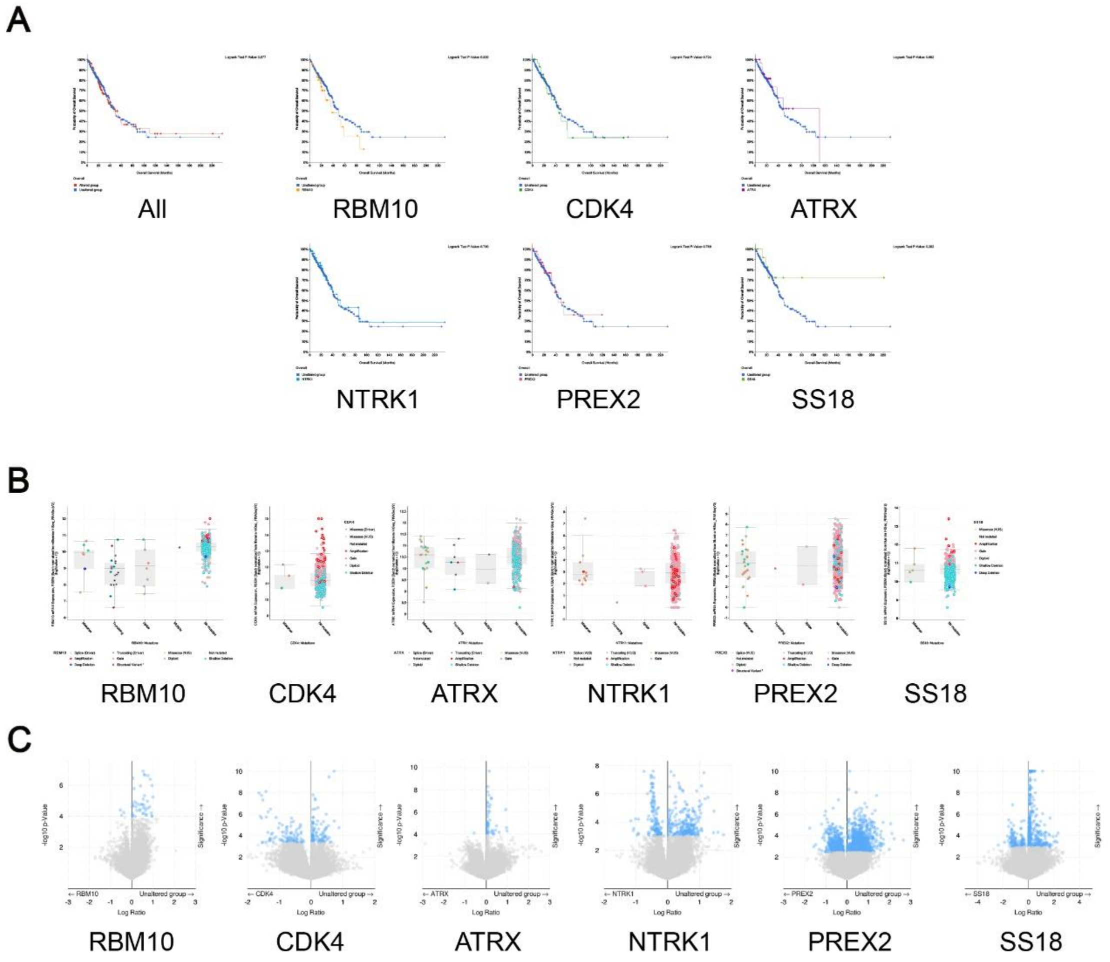

Fig.2 (A) Overall survival of the six gene based on their mutation status. (B) The relationship between SNP and CNV. (C) Differential expressed gene based on the mutation status of each gene.

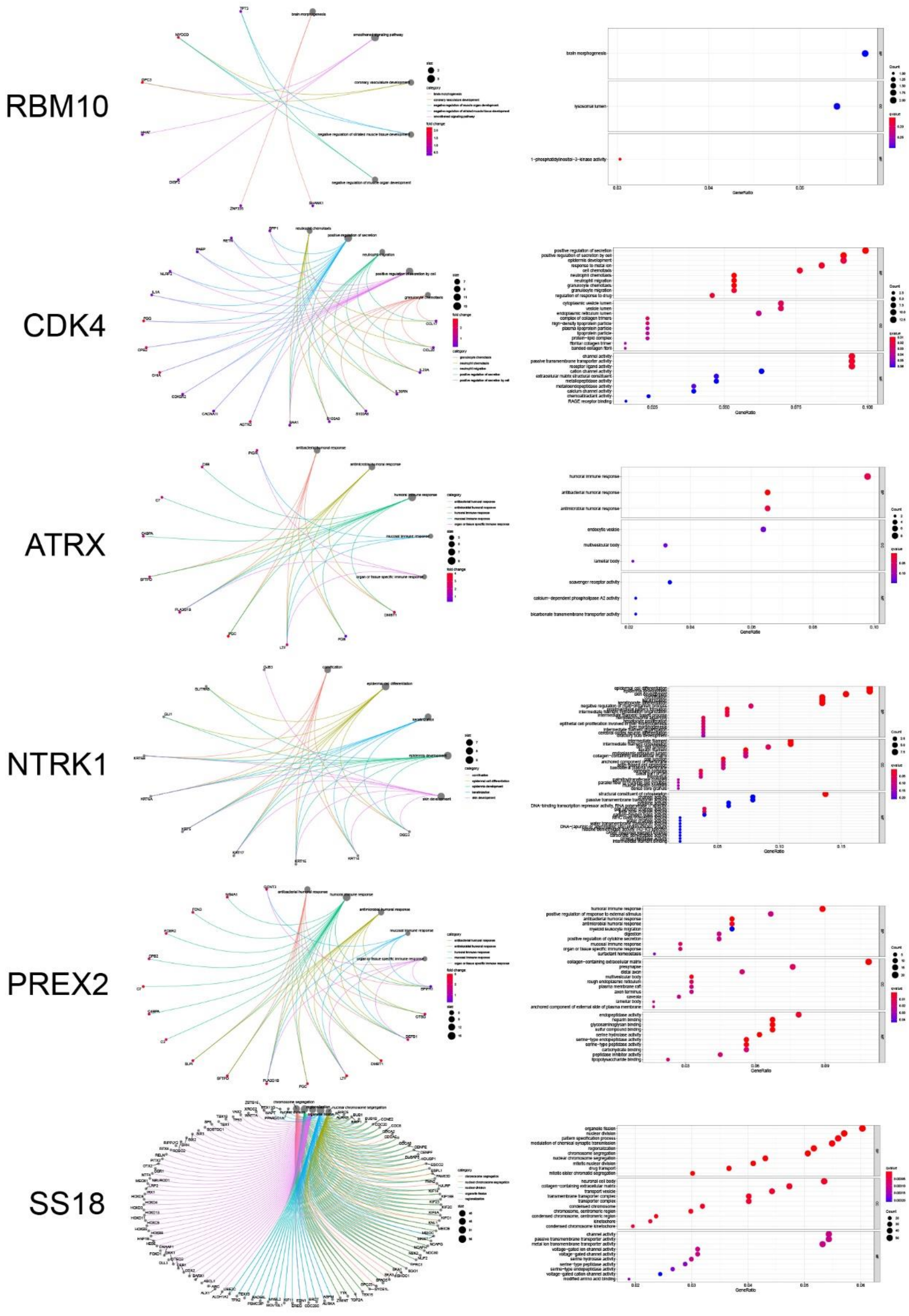

Fig.3 Detail of the GO analysis of the six genes.

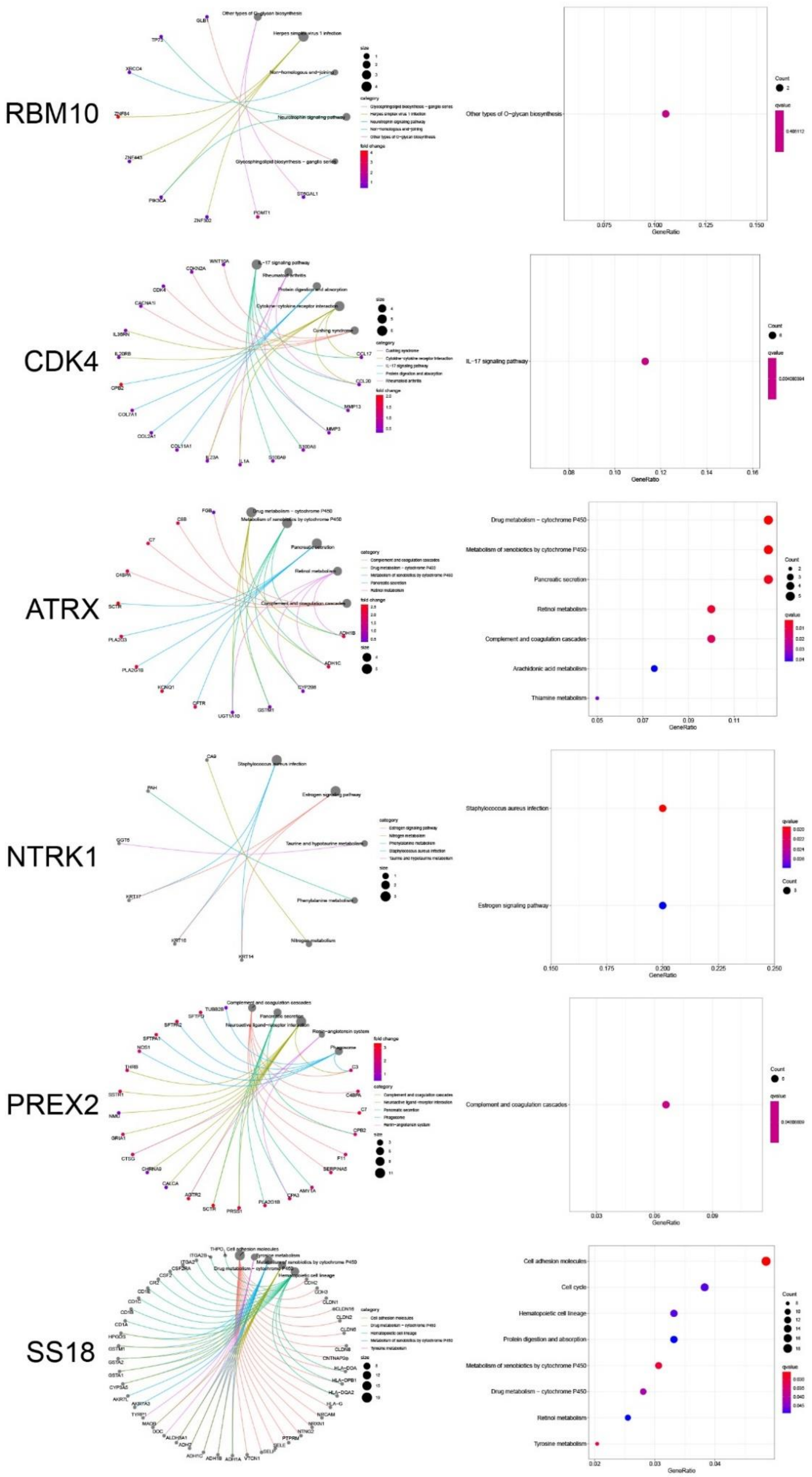

Fig.4 Detail of the KEGG analysis of the six genes.
