# Supplementary material for: Distinct gene mutation profiles among multiple and single primary lung adenocarcinoma
Source: Front Oncol. 2022 Dec 2;12:1014997. doi: 10.3389/fonc.2022.1014997 (PMC9755731; doi:10.3389/fonc.2022.1014997)
Supplement: Supplementary file 3 [file Table_1.docx]

Supplementary table 1. The co-occurrence of gene mutations.

|  | SPLC | | | | MPLC | | | |
| --- | --- | --- | --- | --- | --- | --- | --- | --- |
|  | gene2 | Event Ratio | pValue | Event | gene2 | Event Ratio | pValue | Event |
| EGFR | EPHA3 | 2/103 | 0.0089 | MutEx | ATRX | 2/55 | 0.0443 | MutEx |
|  | KRAS | 0/110 | 0.0000 | MutEx | EPHA5 | 0/57 | 0.0045 | MutEx |
|  | LRP1B | 6/102 | 0.0137 | MutEx | KRAS | 0/61 | 0.0000 | MutEx |
|  |  |  |  |  | LRP1 | 1/56 | 0.0181 | MutEx |
|  |  |  |  |  | LRP1B | 3/56 | 0.0105 | MutEx |
|  |  |  |  |  |  |  |  |  |
| TP53 | RB1 | 12/58 | 0.0009 | Co-Mut | LRP1B | 7/26 | 0.0315 | Co-Mut |
|  |  |  |  |  |  |  |  |  |
| KRAS | FAT3 | 3/14 | 0.0232 | Co-Mut | FAT3 | 3/9 | 0.0252 | Co-Mut |
|  |  |  |  |  | LRP1B | 4/9 | 0.0064 | Co-Mut |
|  |  |  |  |  |  |  |  |  |
| SMARCA4 | GLI2 | 2/10 | 0.0379 | Co-Mut |  |  |  |  |
|  | KMT2C | 2/8 | 0.0211 | Co-Mut |  |  |  |  |
|  | LRP2 | 2/10 | 0.0379 | Co-Mut |  |  |  |  |
|  | TP53 | 6/63 | 0.0122 | Co-Mut |  |  |  |  |
|  |  |  |  |  |  |  |  |  |
| SPTA1 | KMT2D | 4/12 | 0.0018 | Co-Mut | ADGRA2 | 2/4 | 0.0132 | Co-Mut |
|  |  |  |  |  | ATRX | 2/6 | 0.0317 | Co-Mut |
|  |  |  |  |  | FAT3 | 2/7 | 0.0435 | Co-Mut |
|  |  |  |  |  | PRKDC | 2/5 | 0.0216 | Co-Mut |
|  |  |  |  |  |  |  |  |  |
| EPHA3 | LRP1B | 4/15 | 0.0046 | Co-Mut |  |  |  |  |
|  |  |  |  |  |  |  |  |  |
| EPHA5 | LRP2 | 2/10 | 0.0379 | Co-Mut | CDKN2A | 2/5 | 0.0216 | Co-Mut |
|  |  |  |  |  | GLI3 | 2/5 | 0.0216 | Co-Mut |
|  |  |  |  |  | KRAS | 3/6 | 0.0034 | Co-Mut |
|  |  |  |  |  | LRP1 | 3/3 | 0.0006 | Co-Mut |
|  |  |  |  |  | LRP1B | 3/7 | 0.0051 | Co-Mut |
|  |  |  |  |  | TP53 | 4/27 | 0.0289 | Co-Mut |
|  |  |  |  |  |  |  |  |  |
| PREX2 |  |  |  |  | CDKN2A | 2/5 | 0.0216 | Co-Mut |
|  |  |  |  |  | LRP2 | 2/7 | 0.0435 | Co-Mut |

Supplementary Table 2. Genetic screening in sample lineage decomposition.

| Gene name | Variation | DNA change | Variation type |
| --- | --- | --- | --- |
| TP53 | F134S | c.401T>C | SNV |
| TP53 | H193R | c.2369C>T | SNV |
| EGFR | L747_P753delinsS | C.2240_2257del | SNV |
| EGFR | S768_D770dup | C.2303_2311dup | SNV |
| EGFR | T790M | c.2369C>T | SNV |
